# The cancer patient’s friend

**DOI:** 10.3747/co.2007.94

**Published:** 2007-02

**Authors:** J. Bick

The day my friend called me to say that she was discontinuing chemotherapy, I was angry and disillusioned. My first comment was “You can’t! The longer they can keep you alive, the better chance you have of surviving. There are new drugs every day.”

After seven years of remission, Maria’s breast cancer had come back. For almost two years, she had been receiving chemotherapy monthly. It was a rough road, potholed with nausea, heartburn, sleep deprivation, lack of concentration, and intervals of memory loss. Maria’s white blood cell count kept dropping, postponing the chemotherapy sessions. Maria could no longer manage on her own. She needed a caregiver.

On one of my visits, I realized that Maria was in terrible pain. That information came from her caregiver, because Maria never complained. Recent X-rays had showed seven fractures in her spine. Severe osteoporosis had set in. A lot of pain pills were prescribed. Between the pain pills and the sleeping pills, Maria was incoherent most of the time. Her family doctor had prescribed all of this medication; the oncologist seemed interested only in controlling the cancer.

I found a team of doctors able to operate on Maria’s spine. They cemented the fractures to relieve the pain, eliminating the need for all the pain pills. At least now Maria had regained some quality of life.

Two months later, Maria’s oncologist discovered that the chemotherapy cocktail was not working. The cancer was spreading. It had attacked Maria’s lungs, causing pain in her upper back. She was again becoming incoherent, and her balance was off. Her treatment team thought the cancer had reached her brain.

At that point, Maria was changed to weekly treatment, which left her ill most of the time. The smell of food nauseated her, and she began losing weight. Maria was aware enough to realize the changes in her body, and she was rapidly becoming discouraged.

Maria had many questions, but she found it difficult to relate to the doctor. She wanted know if the treatments were helping and what her chances were. I accompanied her to the doctor, prepared to get some answers. She had every right at this point to know where she was heading.

The doctor was very straightforward ... and a little too removed for my liking. He very bluntly told us that, when breast cancer returns, it is fatal. He proposed to continue chemotherapy to keep Maria alive as long as possible.

Maria returned home very sad and disillusioned. That’s when she decided that she could no longer continue treatment. There simply was no hope left.

At her next appointment, Maria told the doctor that there was no point in continuing. She had made up her mind.

The doctor was furious. He said that, if Maria continued treatment, he could keep her alive for another year; however, if she stopped treatment, she would die in a month.

I asked about the quality of life that Maria would have if she continued treatment. The doctor just shrugged his shoulders.

Even I had to admit that there was really no more hope for Maria. I could not be angry with her any longer.

Her first week without treatment, Maria felt a little better. She wasn’t nauseated, but she still couldn’t eat or drink. She found even her usual favourite foods either too salty or too sweet. By the third week, she was bedridden and on intravenous morphine.

With the help of her caregiver, Maria prepared for the end of her life. They made on-call arrangements with the Victorian Order of Nurses, who were wonderful. Maria and her caregiver also found a palliative care doctor who would keep Maria comfortable and pain free.

We were told that a healthy person can live only about three weeks without food or water. Maria fought a cruel war valiantly and with dignity, and she died five weeks after ceasing treatment.

I have to believe that, in the end, she made the right decision.

